# Delineation of motoneuron subgroups supplying individual eye muscles in the human oculomotor nucleus

**DOI:** 10.3389/fnana.2014.00002

**Published:** 2014-02-12

**Authors:** Emmanuel Che Ngwa, Christina Zeeh, Ahmed Messoudi, Jean A. Büttner-Ennever, Anja K. E. Horn

**Affiliations:** ^1^Oculomotor Group, Institute of Anatomy and Cell Biology, Department I, Ludwig-Maximilians-University of MunichMunich, Germany; ^2^German Center for Vertigo and Balance Disorders, Ludwig-Maximilians-University of MunichMunich, Germany

**Keywords:** central caudal nucleus, nucleus of Perlia, extraocular muscles, motoneurons, calretinin, glycine, GABA, eye movements

## Abstract

The oculomotor nucleus (nIII) contains the motoneurons of medial, inferior, and superior recti (MR, IR, and SR), inferior oblique (IO), and levator palpebrae (LP) muscles. The delineation of motoneuron subgroups for each muscle is well-known in monkey, but not in human. We studied the transmitter inputs to human nIII and the trochlear nucleus (nIV), which innervates the superior oblique muscle (SO), to outline individual motoneuron subgroups. Parallel series of sections from human brainstems were immunostained for different markers: choline acetyltransferase combined with glutamate decarboxylase (GAD), calretinin (CR) or glycine receptor. The cytoarchitecture was visualized with cresyl violet, Gallyas staining and expression of non-phosphorylated neurofilaments. Apart from nIV, seven subgroups were delineated in nIII: the central caudal nucleus (CCN), a dorsolateral (DL), dorsomedial (DM), central (CEN), and ventral (VEN) group, the nucleus of Perlia (NP) and the non-preganglionic centrally projecting Edinger–Westphal nucleus (EWcp). DL, VEN, NP, and EWcp were characterized by a strong supply of GAD-positive terminals, in contrast to DM, CEN, and nIV. CR-positive terminals and fibers were confined to CCN, CEN, and NP. Based on location and histochemistry of the motoneuron subgroups in monkey, CEN is considered as the SR and IO motoneurons, DL and VEN as the B- and A-group of MR motoneurons, respectively, and DM as IR motoneurons. A good correlation between monkey and man is seen for the CR input, which labels only motoneurons of eye muscles participating in upgaze (SR, IO, and LP). The CCN contained LP motoneurons, and nIV those of SO. This study provides a map of the individual subgroups of motoneurons in human nIII for the first time, and suggests that NP may contain upgaze motoneurons. Surprisingly, a strong GABAergic input to human MR motoneurons was discovered, which is not seen in monkey and may indicate a functional oculomotor specialization.

## INTRODUCTION

Eye movements are essential for vision, because they direct the fovea to a visual target, and stabilize gaze during locomotion to compensate for head and body movements (Leigh and Zee, 2006; Horn and Leigh, 2011). The motor and premotor pathways for several eye movement types, e.g., saccades and the vestibulo-ocular reflex, are well studied in monkey, and they form the basis for assessing the homologous brain structures in humans, for example, in clinical cases of eye movement disorders (Horn and Leigh, 2011; Kennard, 2011). However, different species have different patterns of eye movements, and different arrangements of their oculomotor subgroups (for review: Büttner-Ennever, 2006). In order to analyze the clinical-anatomical studies involving horizontal and vertical, up- or downward eye movements, the knowledge of the localization of the motoneurons of individual extraocular muscles in human is essential. Despite the fact that efforts on this topic have been undertaken since 1897 (Bernheimer, 1897) in human, the current map of individual motoneuronal groups adopted in most textbooks is still that of the monkey (Warwick, 1953a). In non-human primates, the oculomotor nucleus (nIII) and trochlear nucleus (nIV) lie in the mesencephalic tegmentum at the ventral border of the periaquaeductal gray beneath the aqueduct (for review: Büttner-Ennever, 2006). Since the classical work on the nIII in rhesus monkey by Warwick (1953a) using degeneration techniques, the topographic map has undergone substantial revisions in the primate using retrograde tract-tracing methods (Büttner-Ennever and Akert, 1981; Porter et al., 1983; Büttner-Ennever et al., 2001; Büttner-Ennever, 2006). Neurons supplying the ipsilateral medial rectus muscle (MR) are distributed into three clusters within nIII: the ventral A-group extending into the medial longitudinal fasciclus (MLF), the dorsolateral B-group and the small C-group at the dorsomedial border of nIII (Büttner-Ennever and Akert, 1981). The motoneurons of the ipsilateral inferior rectus muscle (IR) are located dorsally at rostral levels of the nIII, and the motoneurons of the contralateral superior rectus muscle (SR) and ipsilateral inferior oblique muscle (IO) lie partly intermingled within the central nIII of one side (Spencer and Porter, 1981; Porter et al., 1983). The nIV contains only the motoneurons of the contralateral superior oblique muscle (SO; Porter et al., 1983). In primates, a separate midline nucleus at the transition of nIII and nIV, the central caudal nucleus (CCN), contains the motoneurons of the levator palpebrae (LP) muscle, which elevates the upper eyelid (Porter et al., 1989).

The Edinger–Westphal nucleus (EW) lies immediately dorsal to nIII. It is often included in the term “nIII complex,” although it does not contain motoneurons of extraocular muscles. However, recent work has shown that the EW contains different functional cell groups, which must be clearly demarcated from each other and from the nIII proper. In monkey, EW houses the preganglionic (pg) neurons of the ciliary ganglion, in accordance with traditional belief, and is now called EWpg (Horn et al., 2008; May et al., 2008). However, in human, the cytoarchitectural EW represents a cell group of non-pg centrally projecting (cp) neurons that contain urocortin (UCN), and it is therefore now termed EWcp (Horn et al., 2008; Kozicz et al., 2011; Büttner-Ennever and Horn, 2014).

Transmitter content can also distinguish between oculomotor subgroups. Previous studies of transmitter content in cat and monkey have shown that the motoneurons of horizontally moving eye muscles are controlled by glycinergic inputs, whereas those of vertically moving eye muscles by GABAergic afferents (Spencer et al., 1989, 1992; Spencer and Baker, 1992). In addition, more recent reports revealed that only the motoneurons of muscles involved in upgaze, including the LP, are selectively targeted by calretinin (CR)-positive afferents (Ahlfeld et al., 2011; Zeeh et al., 2013); this finding proved very useful in the present study for the recognition of IO and SR motoneurons.

In the experiments reported here, we identified the motoneuron groups of individual eye muscles in human. This was based partly on a comparison with the localization of motoneurons derived from tract-tracing experiments in monkey, and partly on the cytoarchitecture and differential histochemical inputs to motoneuron subgroups revealed by immunocytochemical staining for non-phosphorylated neurofilaments (NP-NF) glutamate decarboxylase (GAD), CR, glycine receptor (GlyR) in human midbrain sections. These groups have also been clearly separated from the EW and the nucleus of Perlia (NP) subgroups, and present a new map of the human oculomotor subgroups. A preliminary version of the map has been published previously (Büttner-Ennever and Horn, 2014).

## MATERIALS AND METHODS

### ANTISERA

#### Choline acetyltransferase

Cholinergic motoneurons were detected with a polyclonal choline acetyltransferase (ChAT) antibody raised in goat (AB144P, Chemicon) against the whole enzyme isolated from human placenta, which is identical to the brain enzyme (Bruce et al., 1985; **Table 1**). In immunoblots, this antibody recognizes a 68–70 kDa protein. The appearance and distribution of ChAT-positive neurons with this antibody in the present study is identical to the data of previous reports (Ichikawa and Shimizu, 1998).

**Table 1:** Sources and dilutions of primary antibodies.

Antigen	Antibody	Host	Antibody source	Dilution
Choline acetyltransferase (ChAT)	Polyclonal anti-ChAT	Goat	Chemicon, Temecula, CA, USA, AB144P	1:100
Calretinin (CR)	Polyclonal anti-CR	Rabbit	SWant, Bellinzona, Switzerland, 7669/3H	1:2500
Urocortin 1 (UCN)	Polyclonal anti-UCN	Rabbit	Sigma, St. Louis, USA, U4757	1:8000
GAD	Monoclonal anti-GAD	Mouse	Biotrend, GC3108	1:4000
α and β subunits of glycine receptor (GlyR)	Monoclonal anti-GlyR (clone mAb4a)	Mouse	Synaptic Systems, Göttingen, Germany, 146 011	1:300
Non-phosphorylated neurofilaments (NP-NF)	Monoclonal anti-NP-NF	Mouse	Sternberger, Lutherville, MD, USA, SMI-32P	1:5000

#### Non-phosphorylated neurofilaments

Non-phosphorylated neurofilaments were detected using a mouse monoclonal antibody (IgG1), supplied as a high titer mouse ascites fluid (**Table 1**). The antibody was raised against homogenized hypothalami recovered from Fischer 344 rats (Sternberger et al., 1982). It reacts with a non-phosphorylated epitope in neurofilament H and is abolished when the epitope is phosphorylated (clone 02-135; SMI32, Sternberger Monoclonals Inc., Lutherville, MD, USA; Sternberger and Sternberger, 1983). This antibody visualizes two bands (200 and 180 kDa) in conventional immunoblots (Goldstein et al., 1987).

#### Glutamic acid decarboxylase

GABAergic terminals were detected with a monoclonal antibody against the GABA-synthetizing enzyme glutamic acid decarboxylase (GAD; GAD_65/67_ GC3108, batch number Z05507, clone 1111, Biotrend, Cologne, Germany; **Table 1**). Two molecular forms of GAD – GAD_65_ and GAD_67_ – are known from different species. There is 65% amino acid sequence homology between the two isoforms. Whereas GAD_67_ is a cytoplasmic protein consisting of 594 amino acid residues, GAD_65_ is an amphiphilic and membrane anchored protein consisting of 585 amino acid residues. The antibody GC 3108 recognizes a linear epitope at the C-terminus of rat GAD, common to both isoforms. The hybridoma secreting the antibody to GAD_65/67_ was generated by fusion of splenocytes from a mouse immunized with fragments of recombinant human GAD_65_ fused to glutathione-*S*-transferase (Ziegler et al., 1996).

#### Glycine receptor

The GlyR is a ligand gated Cl^-^ channel, mediating synaptic inhibition in various brain regions. It is a pentamer consisting of α and β subunits. In this study, a monoclonal mouse antibody, clone mAb4a (Cat. No. 146 011, Synaptic Systems, Göttingen, Germany), was used, which recognizes the α and β subunits of the GlyR (**Table 1**). This antibody results in stronger labeling compared to antibodies directed against the α subunit only (Pfeiffer et al., 1984; Waldvogel et al., 2010). The GlyR is present in postsynaptic structures and intracellular sites involved in protein synthesis and transport shown by electron microscopy studies, which explains the diffuse immunostaining of neuronal somata and punctate labeling along the membranes of neurons (Triller et al., 1985; Baer et al., 2009).

#### Calretinin

A rabbit polyclonal CR antibody (7699/3H, LOT 18299, Swant, Bellinzona, Switzerland) was used to detect CR-containing neuronal profiles (**Table 1**). CR is a calcium-binding protein of the EF-hand family, related to calbindin D-28k and calmodulin, with a widespread distribution within the brain in different species (Andressen et al., 1993; Baizer and Baker, 2006; Baizer and Broussard, 2010). The CR antiserum is produced in rabbits by immunization with recombinant human CR containing a 6-his tag at the N-terminal.

#### Urocortin

For the identification of UCN-containing neurons a polyclonal antibody (Sigma, U-4757; Sigma, St. Louis, USA) was used. It was raised in rabbit using a synthetic peptide corresponding to the C-terminus of human UCN (amino acids 25–40 with N-terminally added lysine), conjugated to keyhole limpet hemocyanin (KLH) as immunogen. The antibody does not cross-react with human or rat corticotrophin releasing factor or human adrenocorticotropic hormone (Bachtell et al., 2003).

### HUMAN TISSUE

The brainstems from seven *postmortem* human cases (case 1 – frozen; cases 2–6 – paraffin embedded) were obtained 24–72 h after death from bodies donated to the Anatomical Institute of the Ludwig-Maximilians-University in accordance with the ethical regulations of the University, and through the Reference Center for Neurodegenerative Disorders of the Ludwig-Maximilians-University with written consent from next of kin, who confirmed the wishes at time of death. All procedures were approved by the Local Research Ethics Committees. The study is in accordance with the ethical standards laid down in the 1964 Declaration of Helsinki. The age of the donators ranged from 54 to 90 years, and there is no history of neurological disease (**Table 2**). The tissue was immersed either in 4% paraformaldehyde in 0.1 M phosphate buffer (PB), pH 7.4, or in 10% formalin for 7 days. Five brainstems were embedded in paraffin, and from each case serial sections of 5, 10, and 20 μm thickness were cut. Sections of 20 μm thickness were used for Nissl- and Gallyas fiber staining, 5 and 10 μm thick sections were immunostained “on-slide” after deparaffination and rehydrating in distilled water. For freeze cutting, one brainstem (case 1) was equilibrated in increasing concentrations of sucrose in 0.1 M PB and cut at 40 μm using a cryostat. Every sixth frozen section (240 μm interval) was defatted, rehydrated, then stained with 0.5% cresyl violet for 5 min. In neighboring sections, the myelin was stained with silver using the physical developing method of Gallyas (Gallyas, 1979). The nomenclature and abbreviations for human brainstem structures are in accordance with the revised new edition of Olszewski and Baxter’s “cytoarchitecture of the human brainstem” (Büttner-Ennever and Horn, 2014).

**Table 2:** Human post-mortem cases used in the study.

Case	Age	Gender	Post-mortem delay (hour)	Fixation duration (day)	Cutting
1	90	Female	24	2	Frozen
2	69	Male	24	2	Paraffin
3	57	Female	24	6	Paraffin
4	67	Male	24	10	Paraffin
5	75	Male	72	10	Paraffin
6	54	Female	24	8	Paraffin

#### Single immunostaining for NP-NF, GAD, CR, UCN

Parallel series of adjacent frozen sections (40 μm) were processed “free-floating,” whereas the paraffin sections (10 μm) were processed “on-slide” after deparaffination in three changes of xylene and rehydration in decreasing concentration of alcohol (100, 96, 90, and 70%) and a final rinse in distilled water. In addition, for the paraffin sections of formalin-fixed tissue an antigen retrieval procedure preceded the protocol for immunostaining: after deparaffinizing, the sections were incubated in 0.01 M sodium citrate buffer (pH 8.5) in a water bath at 80°C for 15 min, and then for another 15 min at room temperature, before being rinsed and started with the immunostaining protocol (Jiao et al., 1999).

After a short rinse in double distilled water and 0.1 M PB, pH 7.4, the sections were treated with 3% H_2_O_2_ and 10% methanol for 15 min to eliminate endogenous peroxidase activity and were washed extensively with 0.1 M Tris-buffered saline (TBS; pH 7.4). To block non-specific binding sites, the sections were then incubated with either 2% normal horse (for NP-NF, GAD, GlyR) or 2% normal goat serum (for CR, UCN) in 0.3% Triton-X 100 in 0.1 M TBS for 1 h at room temperature. Parallel 2 mm spaced series of neighboring sections were subsequently treated either with mouse anti-NP-NF (1:5000; Sternberger) or mouse anti-GAD (1:4000, Biotrend) or mouse anti-GlyR (1:300, Synaptic Systems) or rabbit anti-CR (1:2500, Swant) or rabbit anti-UCN (1:8000; Sigma) for 2 days at 4°C. After washing in 0.1 M TBS, the sections were incubated either in biotinylated horse anti-mouse IgG (1:200; Vector Laboratories) or biotinylated goat anti-rabbit IgG (1:200; Vector Laboratories) at room temperature for 1 h, followed by three washes in 0.1 M TBS. Then, sections were incubated in extravidin-peroxidase (EAP; 1:1000; Sigma) for 1 h at room temperature. After two rinses in 0.1 M TBS, and one rinse in 0.05 M Tris-buffer (TB), pH 8, the EAP complex indicating the antigenic sites was visualized by a reaction in 0.05% diaminobenzidine (DAB) and 0.01% H_2_O_2_ in 0.05 M TB for 10 min. After several rinses in TBS, “free floating” sections were mounted, air-dried, dehydrated in increasing concentrations of alcohol and xylene, and coverslipped in DePex mounting medium (Serva, Heidelberg, Germany).

#### Combined immunoperoxidase labeling for ChAT and GAD

In selected paraffin sections, combined immunoperoxidase labeling was used to simultaneously detect ChAT and GAD.

After deparaffination and rehydration, the sections were washed in 0.1 M TBS (pH 7.4), treated with 1% H_2_O_2_ in TBS for 30 min, were rinsed again, and preincubated with 2% normal rabbit serum in 0.3% Triton-X 100 in TBS for 1 h at room temperature. The sections were then treated with goat anti-ChAT (1:100; Chemicon, AB144P) in TBS with 2% rabbit serum and 0.3% Triton X-100 for 48 h at room temperature. After three washes in 0.1 M TBS, the sections were incubated in biotinylated rabbit anti-goat IgG (1:200, Vector Laboratories) in TBS containing 2% bovine serum albumin for 1 h at room temperature. After three washes in 0.1 M TBS, the sections were treated with EAP (1:1000; Sigma) for 1 h. Then, two rinses with 0.1 M TBS were followed by one wash with 0.05 M TB, pH 8, and the reaction with 0.025% DAB, 0.4% ammonium nickel sulfate, and 0.015% H_2_O_2_ in 0.05 M TB, pH 8, for 10 min. This results in a black staining of ChAT-positive structures. After a thorough washing and blocking of residual peroxidase activity with 1% H_2_O_2_ in 0.1 M TBS, the sections were incubated in 2% normal horse serum in 0.3% Triton-X-100 in 0.1 M TBS for 1 h at room temperature before being transferred to mouse anti-GAD (1:4000; Biotrend, GC 3108) in 2% normal horse serum and 0.3% Triton-X-100 in TBS for 24 h at room temperature. After washing in 0.1 M TBS, the sections were incubated in biotinylated horse anti-mouse IgG (1:200; Vector Laboratories, Burlingame, CA, USA) in TBS containing 2% bovine serum albumin for 1 h at room temperature. The antigen binding site was detected by incubating sections in EAP (1:1000; Sigma, St. Louis, MO, USA) for 1 h and a subsequent reaction with 0.025% DAB and 0.015% H_2_O_2_ in 0.05 M TB (pH 7.6) for 10 min to yield a brown staining of GAD-positive profiles. After washing, the sections were air-dried, dehydrated in alcohol, and coverslipped with DePex mounting medium (Sigma, St. Louis, MO, USA).

### ANALYSIS OF STAINED SECTIONS

The slides were examined with a light microscope Leica DMRB (Bensheim, Germany). Brightfield photographs were taken with a digital camera (Pixera Pro 600 ES, Klughammer, Markt Indersdorf, Germany, or Microfire (Optronics, USA) mounted on the microscope. The images were captured on a computer with Pixera Viewfinder software (Klughammer, Markt Indersdorf) or Picture frame 2.2 (Optronics, USA) and processed with Photoshop 7.0 software (Adobe Systems, Mountain View, CA, USA). The sharpness, contrast, and brightness were adjusted to reflect the appearance of the labeling seen through the microscope. The pictures were arranged and labeled with drawing software (Coreldraw 11.0; COREL).

### QUANTIFICATION OF CR AND GAD INPUTS

The CR and GAD inputs to all motoneuronal groups in nIII and nIV were quantified by counting immunoreactive puncta along the measured length of the contour of a motoneuron with Image J (public domain, Java-based image processing program developed at the National Institutes of Health). The values were transferred in a spreadsheet table for calculation of the statistics (Microsoft Excel, 2010). The analysis of each chosen group was performed on sections from two different cases. In one focus plane, the immunoreactive puncta along the outlines of at least 35 cells in each subgroup were counted. Simultaneous ChAT-immunolabeling was used to identify the motoneurons. Immunoreactive puncta were considered to contact a motoneuron, when its soma and the CR or GAD-positive terminal were in the same focal plane, and no space was seen between them. The ratio of the number of terminals per micrometer of cell outline was calculated with Excel software (Microsoft 2010). Then, the average and mean terminal density of inputs and the standard error of the mean were calculated for all motoneuronal subgroups, including those of the LP.

Data were analyzed with the PRISM 5 software (GraphPad Prism 5, San Diego, CA, USA). Statistical analysis was performed using a one-way analysis of variance (ANOVA). *p* Values below 0.0001 were considered statistically significant. Two groups of downgaze motoneurons were identified: those that receive CR-input and those that do not. In addition, those groups of downgaze motoneurons receiving CR-input were separately analyzed and compared with the CR-input of upgaze motoneurons, using the Bonferroni’s multiple comparison test. *p* Values below 0.05 were considered statistically significant.

## RESULTS

The cytoarchitecture of the nIV and nIII complex was visualized with Nissl- and Gallyas fiber staining, which revealed eight separate cell groups. All these cell groups differed in their staining pattern for the transmitter-related markers GAD, GlyR, and the calcium binding protein CR. These findings are described in detail in the following sections beginning with caudal levels.

### TROCHLEAR NUCLEUS

**FIGURE 1:**
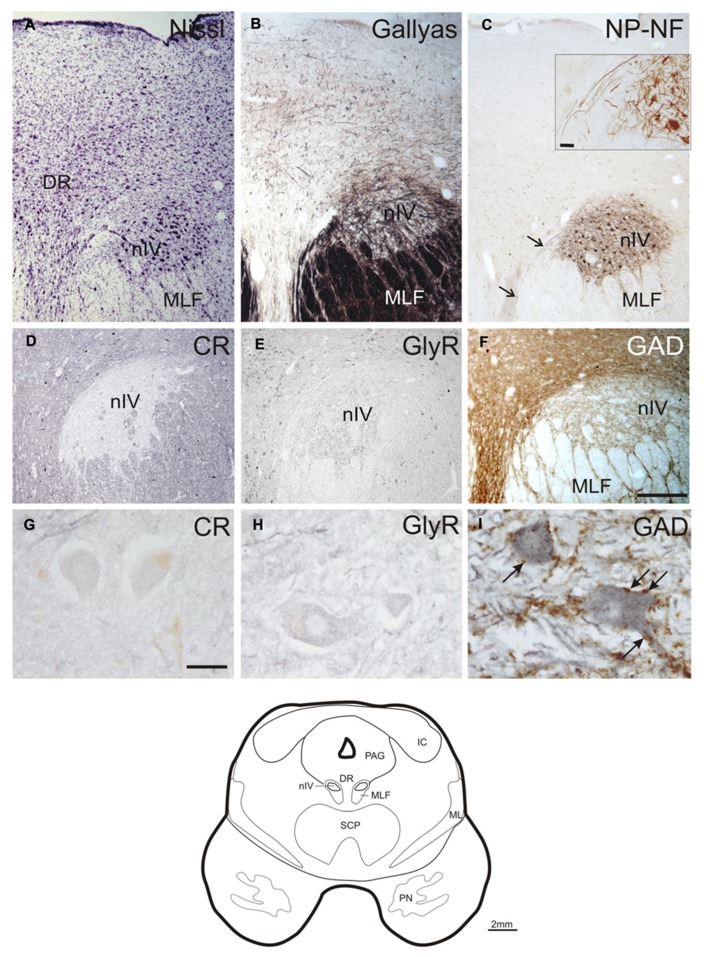
**Transverse sections of the human trochlear nucleus (nIV) demonstrating the cytoarchitecture in cresyl violet (A), Gallyas fiber staining (B), and immunostaining for non-phosphorylated neurofilaments (NP-NF) (C).** The nIV is devoid of calretinin (CR) expressing neurons and fiber profiles **(D,G)**,and it does not express immunoreactivity for the glycine receptor (GlyR) **(E,H)**. The nIV shows a modest supply by GABAergic punctate profiles revealed with antibodies against glutamate decarboxylase (GAD) **(F)**. Panels **(A–C, F)** show neighboring 40 μm frozen sections of one case, panels **(D,E)** show neighboring 10 μm paraffin sections of another case. Panels **(G–I)** are detailed views from **(D–F)**. A line drawing the midbrain section at this level is given at the bottom. DR, dorsal raphe nucleus; IC, inferior colliculus; ML, medial lemniscus; MLF, medial longitudinal fascicle; PAG, periaqueductal gray; PN, pontine nuclei; SCP, superior cerebellar peduncle. Scale bar: **(A–F)** 500 μm; **(G–I,C)** inset 30 μm.

With Nissl- and immunohistochemical staining for NP-NFs, the nIV can be delineated within the mesencephalic tegmentum. At the level of the inferior colliculus (IC), the nIV is clearly outlined as a round nucleus embedded in the fibers of the MLF (**Figures 1A,B**; corresponds to plate 32 in Olszewski and Baxter’s work, 2nd edition, 1982, and 3rd edition by Büttner-Ennever and Horn, 2014). The NP-NF-staining reveals that the dendrites of the motoneurons are interwoven within nIV (**Figure 1C** with inset), and that they are confined to the nucleus at the medial and dorsal aspects. The dendrites extend from the nuclear boundaries at the lateral and ventral aspects and intermingle between the fibers of the MLF. The axons travel medial to the MLF (**Figure 1C**, arrows, inset). As reported by others, two completely separate divisions of the nIV are apparent in the caudo-rostral direction (not shown; Pearson, 1943; Büttner-Ennever and Horn, 2014).

No CR-positive neurons or puncta were found within the boundaries of nIV (**Figures 1D,G**). The same observation was made for GlyR-immunostaining (**Figures 1E,H**). GAD-immunostaining did not reveal any labeled somata within nIV, but numerous labeled puncta were detected around cholinergic motoneurons, many of them most likely representing synaptic terminals (**Figures 1F,I**, arrows).

### CENTRAL CAUDAL NUCLEUS AND CAUDAL OCULOMOTOR NUCLEUS

**FIGURE 2:**
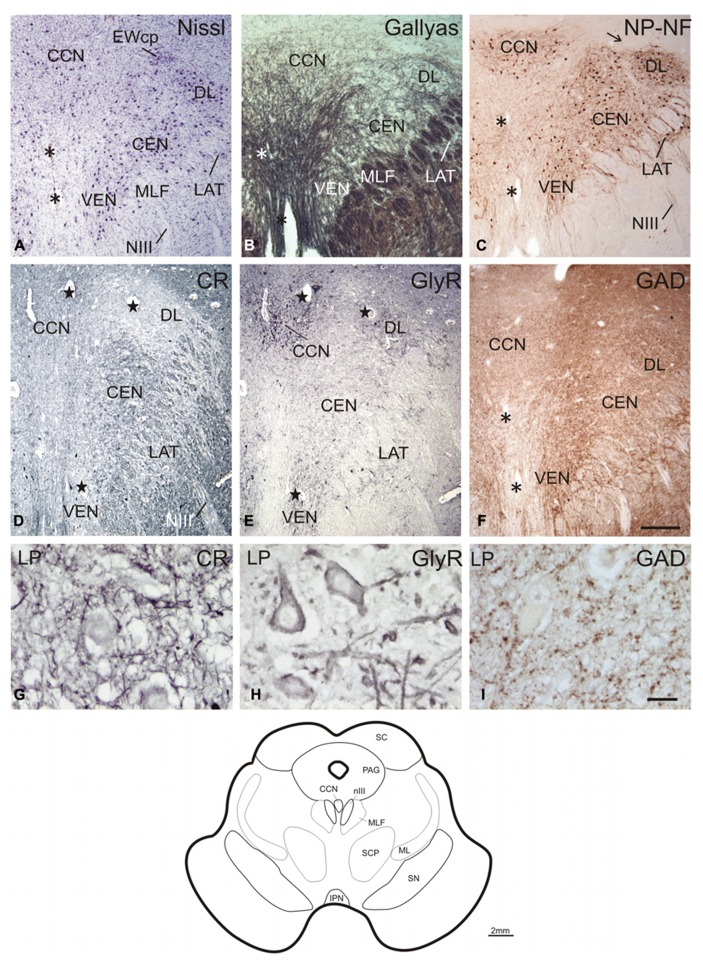
**Transverse sections through the caudal plane of the oculomotor nucleus (nIII).** Cresyl violet **(A)**, Gallyas fiber staining **(B)**, and immunostaining for non-phosphorylated neurofilaments (NP-NF) **(C)** reveal several subnuclei of the oculomotor nucleus complex that exhibit different staining patterns for calretinin (CR) **(D)**, glycine receptor (GlyR) **(E)**, and glutamate decarboxylase (GAD) **(F)**. The central caudal nucleus (CCN) appears as a separate nucleus embedded in the medially descending fibers **(A–C)**. The CCN is high-lighted by its GlyR expression **(E)** and shows a moderate supply by CR- and GAD-positive profiles **(D,F)**. A dorsolateral group (DL) of nIII is separated by encircling fibers **(A–C)**. DL is devoid of CR-positive profiles **(D)**, but rich in GlyR- and GAD-positive profiles **(E,F)**. A similar pattern is seen for the ventral group (VEN) and lateral group (LAT), which forms an island of cells within the medial longitudinal fascicle (MLF) **(A–F)**. A central group (CEN) is high-lighted by its strong expression of CR **(D)**, but shows less staining for GAD **(F)** and almost none for GlyR **(E)**. Panels **(A–C,F)** show neighboring 40 μm frozen sections of one case, **(D,E)** neighboring 10 μm paraffin sections of another case. Panels **(G–I)** are detailed views from **(D–F)**. Asterisks label corresponding blood vessels in neighboring frozen sections of one case **(A–C,F)**, stars label those in neighboring paraffin sections from a different case **(D,E)**. Detailed views of levator palpebrae (LP) motoneurons in CCN are shown for CR **(G)**, GlyR **(H)**, and GAD-immunoreactivity **(I)**. A line drawing at the bottom shows the midbrain section at this level. IPN, interpeduncular nucleus; ML, medial lemniscus; PAG, periaqueductal gray; SC, superior colliculus; SCP, superior cerebellar peduncle; scale bars: **(A–F)** 500 μm; **(G–I)** 30 μm.

The caudal end of nIII appears as a V-shaped nucleus with the CCN dorsally embedded in the V-opening shown on a plane approximately 2 mm further rostral to nIV (**Figures 2A–C**; corresponds to plate 34 in Büttner-Ennever and Horn, 2014). At this plane, a small group of densely packed neurons adjacent to the dorsal rim of nIII becomes apparent in Nissl-stained sections. This cell group consists of UCN-positive neurons (Ryabinin et al., 2005; Horn et al., 2008) and has recently been termed EWcp (**Figure 2A**; Kozicz et al., 2011). As shown earlier, the EWcp does not express NP-NF-immunoreactivity (**Figure 2C**, arrow; Horn et al., 2008). Within the main nIII, four subgroups can be delineated at this level: a ventral (VEN) group outlined dorsomedially by traversing fibers shown by Gallyas fiber staining (**Figure 2B**), and a central (CEN) group dorsal to it (**Figures 2A,C**). A lateral (LAT) group is apparent as cell islands between the rootlets of the third nerve (NIII), separated from the main nucleus by the traversing fibers of the MLF (**Figures 2A–C**). A dorsolateral group (DL) appears as a relatively isolated circular subnucleus, most apparent in Gallyas staining and NP-NF-immunostaining (**Figures 2B,C**).

**FIGURE 3:**
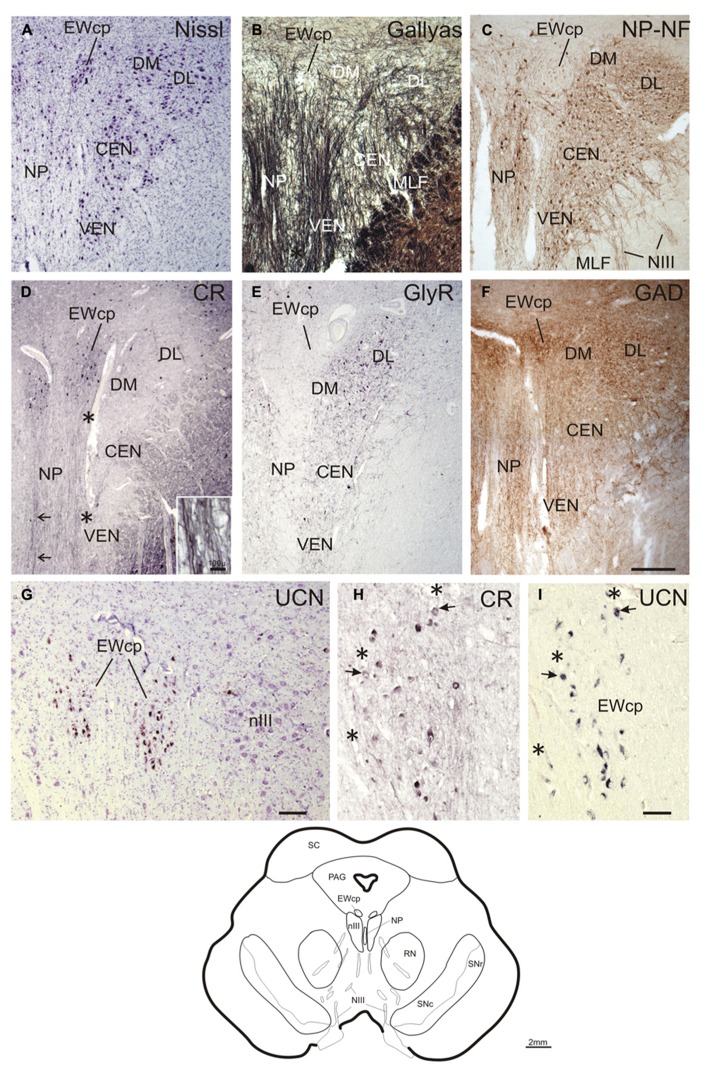
**Transverse sections at the level of the mid oculomotor nucleus (line drawing at the bottom).** Cresyl violet **(A)**, fiber staining **(B)**, and immunostaining for non-phosphorylated neurofilaments (NP-NF) **(C)** reveal six subnuclei of the nIII complex at this level that exhibit different staining patterns for calretinin (CR) **(D)**, glycine receptor (GlyR) **(E)**, and glutamate decarboxylase (GAD) **(F)**. The nucleus of Perlia (NP) forms an elongated midline cell group separated from the main nucleus by dorsoventrally traversing fibers **(A,B)**, some expressing CR-immunoreactivity (**D**, arrows, inset). At the dorsomedial border of nIII, a compact cell group forms the centrally projecting non-preganglionic part of the Edinger–Westphal nucleus (EWcp), which contain urocortin (UCN)-positive and some scattered CR-positive neurons **(D,G)**. The EWcp does not contain NP-NF **(C)**, is devoid of GlyR **(E)**, but receives a strong GAD input **(F)**. High power magnification of two adjacent 5 μm paraffin sections immunostained for CR and UCN reveal only few UCN-positive neurons expressing CR (**H,I**, arrows). Corresponding blood vessels are indicated by asterisks. PAG, periaqueductal gray; RN, red nucleus; SC, superior colliculus; SN, substantia nigra (reticulata and compacta). Scale bar: **(A–F)** 500 μm; **(G)** 100 μm; **(H,I)** 50 μm.

**FIGURE 4:**
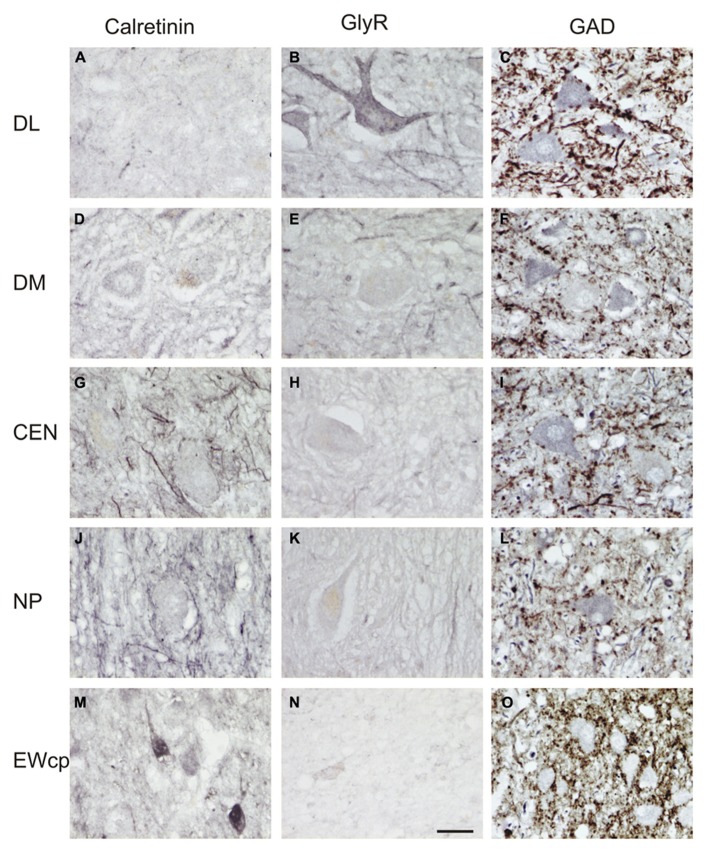
**High-power photographs of the staining pattern with different markers in the oculomotor nucleus complex.** Numerous calretinin (CR)-positive profiles are found in association with cell bodies only in CEN and NP **(G,J)**, but not in DL, DM **(A,D)**. CR-staining of somata is found in EWcp (**M**; see also **Figure 3**). Strong labeling for glycine receptors (GlyR) is found only in the dorsolateral group (DL) **(B)**, and with some traversing fibers in the dorsomedial group (DM) **(E)**, but none in CEN, NP and EWcp **(H,K,N)**. Different density of glutamate decarboxylase (GAD)-positive puncta is seen around choline acetyltransferase (ChAT)-immunoreactive motoneurons in the dorsolateral (DL) **(C)**, dorsomedial (DM) **(F)**, central groups (CEN) **(I)**, and the nucleus of Perlia (NP) **(L)**. The strongest supply by GAD-positive punctate profiles is present around ChAT-negative non-preganglionic centrally projecting neurons in the Edinger–Westphal nucleus (EWcp) **(O)**. Scale bar **(A–O)** 30 μm.

A strong supply by CR-positive fibers and nerve endings was evident in the CEN group, thereby highlighting it selectively from CR-negative DL and DM groups (**Figures 2D** and **3D**; **Figures 4A,D**). A considerable supply was also found around the LP motoneurons in the CCN (**Figures 2D,G**). Immunostaining for the GlyR revealed a strong signal in the CCN (**Figure 2E**). At high magnification, the GlyR-immunostaining appears as diffuse staining of the neuronal somata and punctate labeling along the neuronal membrane surface of somata and dendrites of LP motoneurons (**Figure 2H**). Within the nIII, the DL and VEN subgroups were highlighted by their strong GlyR-immunostaining (**Figure 2E**). As in nIV, a strong supply by GAD-immunopositive puncta was evident in CCN and in all subgroups of the caudal nIII (**Figures 2F,I**). The DL and VEN subgroups were outlined by their relatively stronger abundance of GAD-positive punctate labeling compared to other subgroups (**Figure 2F**).

### MID nIII, NUCLEUS OF PERLIA

At planes through the nIII 2 mm further rostral (corresponding to plate 36, Büttner-Ennever and Horn, 2014), the medial portion of the EWcp appears between the dorsal parts of nIII (**Figures 3A–F**). The NP-NF-negative EWcp is embedded in dorsoventrally traveling fibers (**Figures 3B,C**). At the midline of this level, an unpaired cell group is separated from the main nIII by dorsoventrally traversing fibers. This nucleus is called the nucleus of Perlia (NP) (**Figures 3A–C**; Perlia, 1889). Between EWcp and the DL group, an additional dorsomedial group (DM) appears at this level (**Figures 3A–F**).

Calretinin-immunostaining revealed only a few scattered small CR-positive neurons in nIII, mainly at the dorsomedial and medial border between both nIII. A group of CR-positive neurons is present in the dorsal, medial, and ventral perioculomotor region, in part covering the EWcp (**Figures 3D** and **4M**). The careful analysis of neighboring 5 μm thick paraffin sections, stained either for CR or UCN (**Figures 3G–I**), revealed that both populations do not overlap to any great extent. Only few UCN-positive neurons in EWcp express CR-immunoreactivity (**Figures 3H,I**, arrows). The CR-positive neurons in EWcp may form the origin of at least one portion of the dorsoventrally running fibers that embrace the NP and separate it from the lateral nIII (**Figure 3D**, arrows, insert). As for CCN and CEN, a considerable supply of CR-positive axonal profiles was found around neurons in NP (**Figures 3D** and **4G,J**).

Whereas CEN, NP, and EWcp were largely devoid of GlyR-positive neuronal profiles (**Figures 3E** and **4H,K,N**), the DM expressed some GlyR-immunoreactivity in addition to DL and VEN (**Figures 3E** and **4B,E**). The GlyR-labeling of DM most probably represents dendrites of the adjacent motoneurons of LP and the DL group, which are strongly labeled (**Figures 4B,E**). The close inspection of sections stained for ChAT and GAD revealed that in all nIII subgroups the somata and proximal dendrites of the cholinergic motoneurons were associated with GAD-immunoreactive profiles. A similar strong GAD-input was found in the DL, CEN, VEN subgroups, as in the NP (**Figures 4C,I,L**). The DM, LAT showed the weakest supply from GAD-positive puncta, the non-cholinergic neurons in EWcp the strongest (**Figures 4F,O**).

### ROSTRAL nIII

**FIGURE 5:**
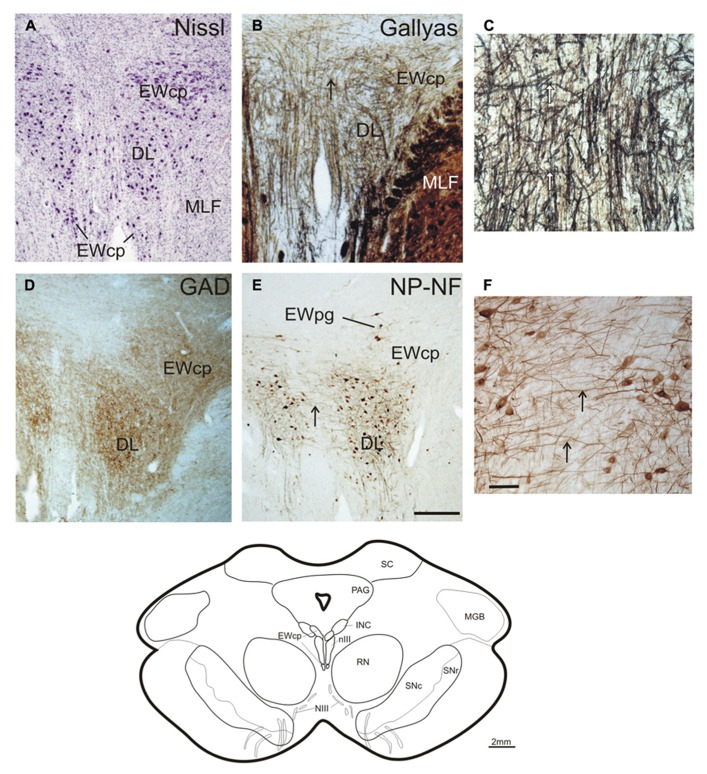
**Transverse sections at the level of the rostral oculomotor nucleus (line drawing at the bottom).** Cresyl violet **(A)**, Gallyas-fiber staining **(B)** with detail **(C)**, immunostaining for glutamate decarboxylase (GAD) **(D)**, and non-phosphorylated neurofilaments (NP-NF) **(E)** with detail **(F)**. Note, unlike the preganglionic neurons of the Edinger–Westphal nucleus (EWpg), the centrally projecting non-preganglionic neurons of EWcp do not express non-phosphorylated neurofilaments (NP-NF) **(C)**. The strong labeling of GAD-positive puncta in the most rostral group in the nIII speaks for a continuation of the dorsolateral group (DL), which most likely corresponds to the medial rectus B-group in monkey **(D)**. Note the presence of numerous crossing fibers between both nIII at this level (**B,C,E,F**, arrows). INC, interstitial nucleus of Cajal; MGB, medial geniculate body; PAG, periaqueductal gray; RN, red nucleus; SN, substantia nigra (reticulata and compacta). Scale bar: **(A,B,D,E)** 500 μm; **(C,F)** 100 μm.

Another 2 mm further forward, at the rostral end of nIII, the DL is the only remaining subgroup. It is bordered by the EWcp, which forms a large cell group dorsally and a small extension ventrally (**Figure 5A**; corresponding to plate 38, Büttner-Ennever and Horn, 2014). Interestingly at this level fibers arising from the nIII of both sides intermingle intensely with each other, apparent from Gallyas staining and NP-NF-immunostaining (**Figures 5B,C,E,F**, arrows). GAD-positive puncta covered the DL and EWcp densely (**Figure 5D**).

### QUANTITATIVE ANALYSIS OF GAD AND CR-POSITIVE INPUTS

**FIGURE 6:**
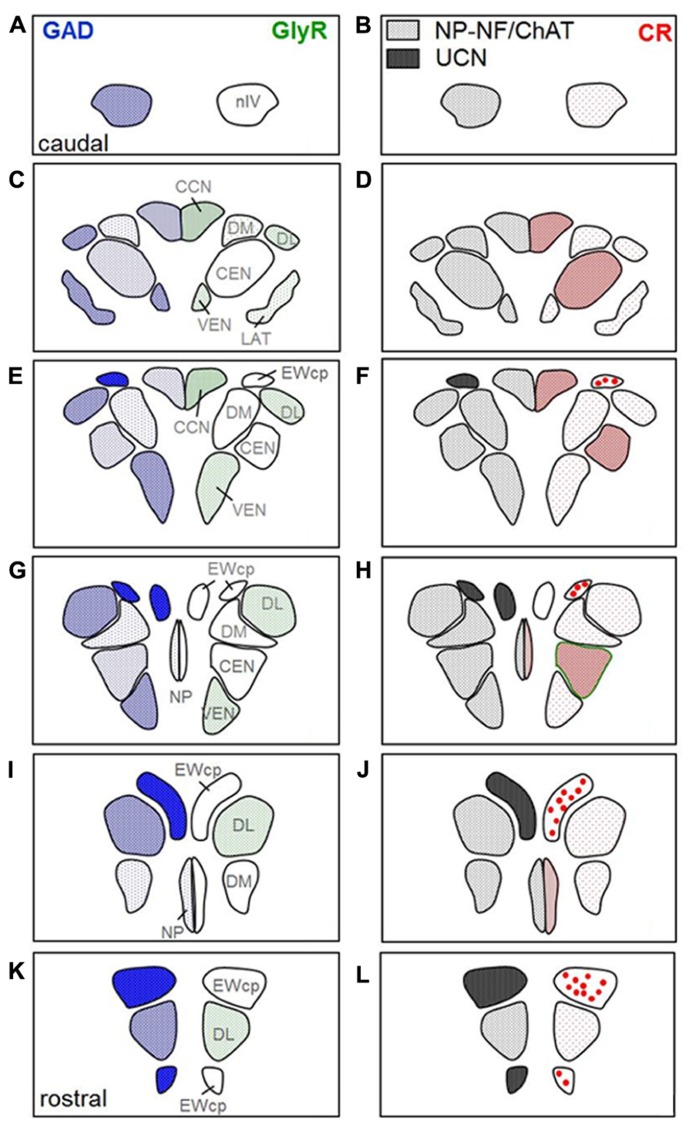
**Two parallel series of drawings of transversal sections through the trochlear (nIV) and oculomotor nucleus (nIII) complex arranged from caudal to rostral.** Panels **(A,B)** correspond to the level of **Figure 1**, **(C,D)** to the level of **Figure 2**, **(G,H)** to the level of **Figure 3**, and **(K,L)** to the level of **Figure 5**. Panels **(E,F** and **I,J)** correspond to additional intermediate levels. On the left, the density of glutamate decarboxylase (GAD) positive puncta and the intensity of immunostaining for the glycine receptor (GlyR) in the different subgroups is indicated by blue hatching on the left and green hatching on the right, respectively. The right column shows the presence of neurons expressing immunoreactivity for urocortin (UCN, black), non-phosphorylated neurofilaments (NP-NF; gray) and calretinin (CR; red filled circles). The density of CR-positive profiles is indicated by different grades of red hatching.

**FIGURE 7:**
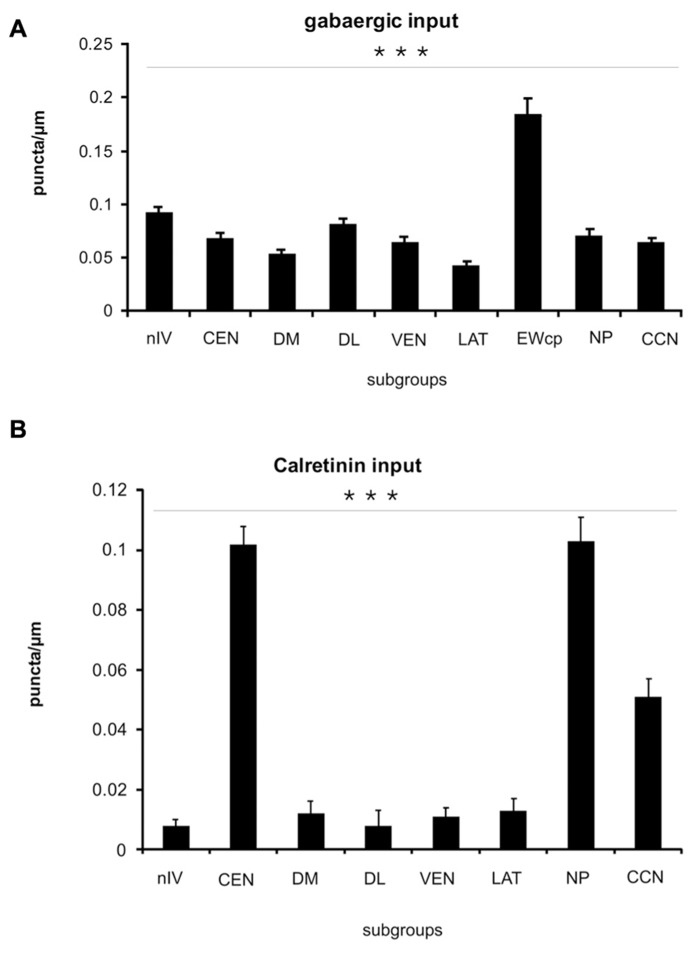
**Histogram of the quantitative analysis of GABAergic and calretinin (CR) input to motoneurons in the oculomotor and trochlear nucleus.** The values are given in **Table 3.** **(A)** The GABA-input was quantified by counting glutamate decarboxylase-positive (GAD) puncta along the measured length of the contour of a given neuron. The mean terminal density of input and the standard error of the mean values were calculated for each subgroup. The one-way analysis of variance revealed a significant difference (*p* < 0.001). The strongest input is seen to non-preganglionic centrally projecting neurons in the Edinger–Westphal nucleus (EWcp; compare to **Figure 4O**), whereas the neurons of all other subgroups did not show major differences. **(B)** The strongest CR input is seen to neurons of the nucleus of Perlia (NP), the central group in oculomotor nucleus (CEN), and the central caudal nucleus (CCN). The number of counted CR-positive puncta associated with putative upgaze motoneurons is significantly higher compared to those around horizontal – and downgaze motoneurons (***p < 0.001).

A summarized view of the histochemical properties is given in **Figure 6**. For verification of the impression received from visual inspection, the GAD- and CR-positive inputs were quantified by counting immunoreactive puncta along the outlines of the perimeter of somata and proximal dendrites in all subgroups of nIII and nIV. The quantitative analysis of GAD-positive puncta confirmed the visual impression, and revealed that the strongest GABAergic input was found to the somata of EWcp neurons (**Figures 6C,E,G,I,K** and **7A**; see also **Figure 4O**) with an averaged density of 0.183 puncta/μm (see **Table 3**). Similar strong GAD-input was found to the motoneurons in nIV, CEN, DL, VEN, NP, and CCN (**Table 3**; **Figures 6A,C,E,G,I,K** and **7A**). The weakest supply was found to involve motoneurons in the DM and LAT subgroups (**Table 3**; **Figures 6A,C,E,G,I,K** and **7A**). The one-way ANOVA revealed a significant difference of the mean values with *p* < 0.001 (**Figure 7A**). GlyR-immunostaining was only found in CCN and the DL and VEN subgroups in nIII, all with a similar intensity (**Figures 6C,E,G,I,K**). All motoneurons including neurons of NP expressed ChAT- and NP-NF-immunostaining, the neurons in EWcp contain UCN, as already shown previously (Horn et al., 2008; **Figures 6B,D,F,H,J,L**).

**Table 3:** Quantification of calretinin and GABAergic input to nIV and nIII subgroups.

	CR		GAD	
Subgroup	Puncta/μm	SE of mean	Puncta/μm	SE of mean
nIV	0.008	0.002	0.091	0.006
CEN	0.102	0.006	0.067	0.006
DM	0.012	0.004	0.052	0.005
DL	0.008	0.005	0.08	0.006
VEN	0.011	0.003	0.063	0.006
LAT	0.013	0.004	0.041	0.005
NP	0.103	0.008	0.069	0.008
CCN	0.051	0.006	0.063	0.005
EWcp	0	0	0.183	0.016

As is apparent from visual inspection of the immunocytochemical staining, the strongest CR-input is found around neurons of the NP, and around motoneurons in CEN (**Table 3**; **Figures 6D,F,H,J** and **7B**). Furthermore, a high density of CR-positive puncta was noticed in the CCN with 0.051 puncta/μm (**Table 3**; **Figures 6D** and **7B**). In contrast, only a few motoneurons in all other motoneuronal subgroups were associated with CR-positive profiles at an average density of around 0.01 puncta/μm (**Table 3**; **Figure 7B**). A comparative analysis revealed that the density of CR-positive puncta around motoneurons for upgaze in CCN and the CEN subgroup was significantly stronger than those around motoneurons for down- or horizontal gaze (*p* < 0.001). Even those down- and horizontal gaze motoneurons receiving some CR-input were contacted by significantly less CR-positive puncta, when separately analyzed and compared with the CR-input of upgaze motoneurons (Bonferroni’s multiple comparison test; *p* < 0.05; not shown).

## DISCUSSION

In this study of the histochemical characteristics of the human nIII and nIV, eight cell groups were distinguished from each other. From these results, and those of a previous study on non-human primates (Zeeh et al., 2013), a map of the subgroups of the human nIII is drawn up here, proposing the target of innervation, for each individual subgroup. In the following sections, the subgroups will be discussed in terms of their proposed function.

### OCULOMOTOR SUBGROUPS INVOLVED IN UPGAZE

#### Motoneurons of superior rectus and inferior oblique muscles

**FIGURE 8:**
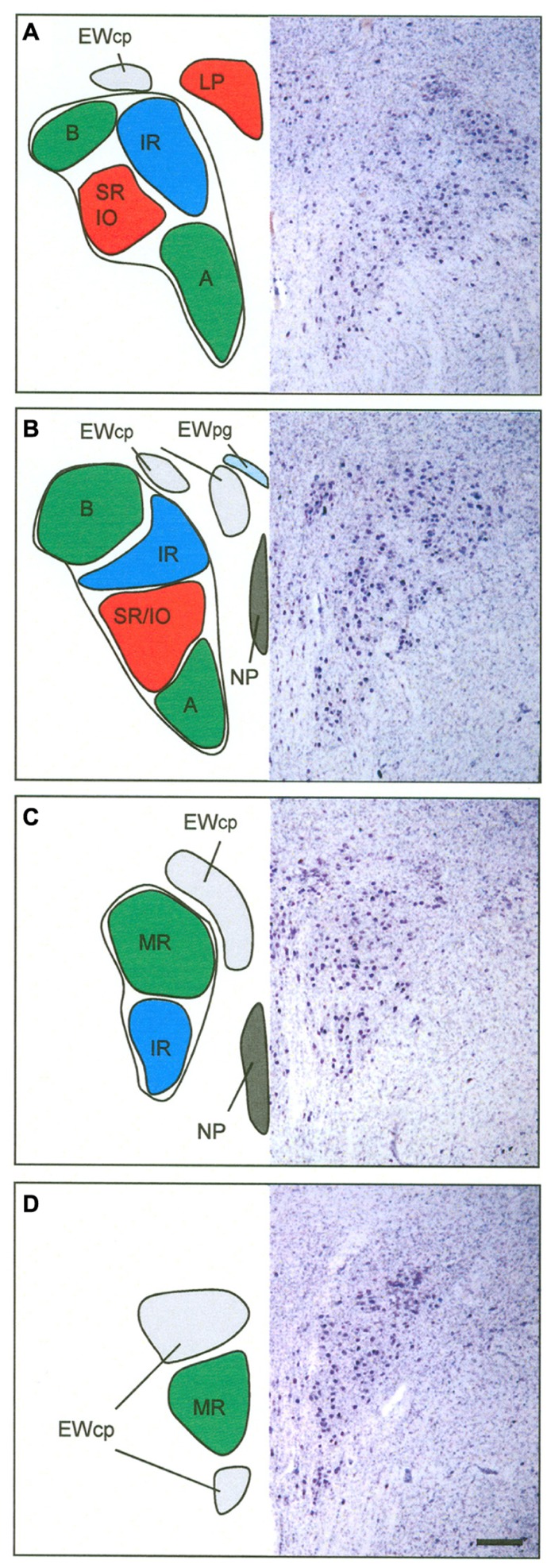
**Proposed map of the motoneurons for individual extraocular muscles in human shown at four representative planes from caudal to rostral.** The right half shows corresponding sections in Nissl staining to demonstrate the cytoarchitecture. The central caudal nucleus at most caudal planes contains the motoneurons of the levator palpebrae muscle (LP) **(A)**. The medial rectus muscles (MR) is represented in two groups, the dorsolateral B- and the ventral A-group **(A,B)**. The central group represents the motoneurons of the inferior oblique (IO) and superior rectus (SR) muscle **(A,B)**. The nucleus of Perlia (NP) is separated from the main nucleus, but may contain SR motoneurons as well **(B,C)**. The dorsomedial group corresponds to the inferior rectus motoneurons (IR) **(A–C)**. The centrally projecting neurons of the Edinger–Westphal nucleus (EWcp) appear as a single lateral group on caudal levels dorsal to nIII **(A)**, adjoined by a medial group further rostrally **(B)**, which both merge to a single dorsal group **(C)**. Another ventral extension of the EWcp appears on rostral levels **(D)**. Note that the preganglionic neurons in the EWpg do not form a compact nucleus **(B)**. Scale bar: **(A–D)** 500 μm.

As in monkey, only selected subgroups within nIII receive a strong input from CR-positive afferents (Zeeh et al., 2013); in human these include the CCN, the CEN group, and NP. Combined tract-tracing and CR-immunostaining experiments in monkey have shown that the CR-positive input was confined to motoneurons participating in upgaze, e.g., SR, IO, and LP in the CCN (Fuchs et al., 1992; Zeeh et al., 2013). Furthermore, tracer injections into the IO or SR muscles in monkey revealed that these two subgroups occupy a similar portion in the central part of the caudal nIII, except for the fact that the SR motoneurons are located contralaterally and tend to lie more medially to the IO motoneurons, which project to the ipsilateral eye muscle. The dendrites of retrogradely labeled IO and SR motoneurons are intimately intermingled and are not confined to any individual cytoarchitectural borders (Spencer and Porter, 1981; Zeeh et al., 2013). Based on the similar anatomical and histochemical features of the CEN group including the selective CR input, the CEN subgroup in the human nIII is considered as the location of SR and IO motoneurons (**Figure 8**).

With combined tract-tracing studies, three sources of the CR input to the nIII complex have been identified in monkey: the rostral interstitial nucleus of the medial longitudinal fascicle (RIMLF), the interstitial nucleus of Cajal (INC) and the y-group of the vestibular nuclei (Ahlfeld et al., 2011). The RIMLF contains premotor neurons of different types; some exhibit a high-frequency burst for upward saccades, others for downward saccades, and they are all intermingled with each other (Büttner et al., 1977; Horn and Büttner-Ennever, 1998). Considering their targets, the CR-positive population probably represents the premotor burst neurons for upward saccades (Ahlfeld et al., 2011). The CR-input from INC to upgaze motoneurons may derive from premotor burst-tonic neurons involved in integration of the velocity signal from RIMLF into the eye-position signal, required for gaze stabilization after a saccade (Fukushima et al., 1992). The lack of GAD in CR-immunopositive neuronal profiles in monkey nIII as revealed by double-immunofluorescence and confocal scanning, indicated that the CR input is excitatory (Zeeh et al., 2013). CR-positive projections from the y-group to SR and IO motoneurons may provide the excitatory drive during smooth pursuit eye movements (Partsalis et al., 1995). The functional significance of the selective CR presence in upgaze pathways remains unclear, it has been discussed in previous publications (Ahlfeld et al., 2011; Zeeh et al., 2013).

#### Central caudal nucleus

Panegrossi (1898), was the first to describe the CCN in human. Originally he had termed the nucleus on the midline, situated between the oculomotor nuclei at caudal levels, as *nucleus posterior dorso-centralis*. He found this nucleus as a constant feature in human, and noted it also in monkey, dog, and cat. Since this nucleus degenerated after removal of the bulbus in cat, he designated it as part of the nIII (review: Warwick, 1953a,b). Similarly, Tsuchida (1906) described a central medial nucleus between the main cell columns of caudal nIII, but he did not relate it to Panegrossi’s findings. He called this medial nucleus the *caudal central nucleus* (Tsuchida, 1906). In spite of the fact that Tsuchida (1906) designated it probably to the dorsal raphe nucleus, his term was later adopted for the midline nucleus containing LP motoneurons. Based on removal of individual eye muscles in monkey, Warwick was the first to show that the CCN contains the LP motoneurons (Warwick, 1953b). This was later confirmed with tract-tracing methods, also showing that the LP motoneurons of both eyes are intermingled within the CCN, with a slight predominance for a contralateral representation (Porter et al., 1989). There are conflicting reports as to whether some LP motoneurons innervate the muscles of both sides (Sekiya et al., 1992; Van der Werf et al., 1997), or whether LP populations are completely separated for each eye (Porter et al., 1989). As in monkey, the CCN in human forms an unpaired nucleus dorsal to the caudal end of nIII (Schmidtke and Büttner-Ennever, 1992; Horn and Adamcyzk, 2011; Büttner-Ennever and Horn, 2014). Furthermore, the present study revealed, that in addition to a significant CR-input, there is a strong input from GABAergic and glycinergic afferents to LP motoneurons, as found in monkey (Horn and Büttner-Ennever, 2008; Zeeh et al., 2013). One possible source of direct or indirect inhibitory GABAergic afferents is the nucleus of the posterior commissure, since lesions of this area result in lid retraction (Schmidtke and Büttner-Ennever, 1992; Averbuch-Heller, 1997). A further direct inhibitory connection was shown from pontine neurons at the rostral and ventral border of the principal trigeminal nucleus to LP motoneurons in the CCN, which presumably provide the inhibition during blinks (May et al., 2012). The glycinergic input to LP motoneurons may originate from saccadic omnipause neurons, as indicated by tract-tracing studies in monkey (Horn and Büttner-Ennever, 2008). The function of this connection is not clear, yet, but may contribute to pathways involved in blink-saccade interaction (Leigh and Zee, 2006).

Premotor neurons in the medial RIMLF in cat, and in the M-group in monkey, which target LP motoneurons, represent a further possible CR source (Horn et al., 2000; Chen and May, 2002). Furthermore, a monosynaptic excitatory connection from INC to LP motoneurons has been described in cat (Chen and May, 2007). This projection may originate from the same premotor neurons in INC, which target SR and IO motoneurons, thereby coupling vertical eye and lid movements not only during saccades, but also during gaze holding, to provide a larger, and freer upper field of vision.

#### Nucleus of Perlia

The NP was described by Perlia (1889) and originally considered as a cell group participating in the control of convergence, but up to now, without any proof (Warwick, 1955). In spite of several references describing the presence of the NP in non-human primates as a labeled midline group, after tracer injections into the ciliary ganglion (Burde, 1983, 1988; Burde and Williams, 1989; Ishikawa et al., 1990), its existence is still questioned in these species. The tracer labeled neurons are more likely to represent motoneurons of multiply-innervated muscles fibers (MIF) of the IO and SR, due to superficial contamination of the muscles as discussed previously (Büttner-Ennever et al., 2001; Horn et al., 2008). In fact, the morphology of the neurons of NP and their histochemical properties, e.g., expression of ChAT, cytochrome oxidase, NP-NF, and chondroitin sulfate proteoglycans, suggest that they may present motoneurons of singly-innervated twitch muscle fibers (SIF; Eberhorn et al., 2005; Horn et al., 2008). The present study demonstrated a CR input to NP and thereby indicates a role in upgaze, which supports the hypothesis that NP may represent SR twitch motoneurons that are separated from the main subgroup in nIII by dorsoventrally traveling nerve fibers (Horn et al., 2008; Büttner-Ennever and Horn, 2014).

### OCULOMOTOR SUPGROUPS INVOLVED IN DOWNGAZE

#### Motoneurons of superior oblique and inferior rectus muscles

In addition to the SO motoneurons in nIV, the IR motoneurons in nIII participate in downward eye movements (Leigh and Zee, 2006). In monkey, the IR motoneurons lie within the rostral half of nIII appearing medial to the B group of the MR motoneurons. At the rostral end of nIII they form the dorsal part of nIII (Evinger, 1988; Büttner-Ennever, 2006). Unlike motoneurons of horizontal moving eye muscles, a strong GABAergic input was found to the motoneurons of all vertically pulling eye muscles in monkey, including those for downgaze (Spencer and Baker, 1992). One well known source for GABAergic afferents to the vertically pulling eye muscles arises from the secondary vestibulo-ocular neurons in the superior vestibular nuclei (de la Cruz et al., 1992; Wentzel et al., 1996; Highstein and Holstein, 2006). Electrophysiological and pharmacological studies have shown that stimulation of the vestibular nerve results in inhibitory postsynaptic potentials in the ipsilateral nIV and nIII, which are blocked after administration of GABA antagonists (Obata and Highstein, 1970). Similarly, a lesion of the MLF results in a drastic decrease of GABA in nIII and nIV in cat (Precht et al., 1973).

Another source for GABAergic afferent input to nIII and nIV is the INC. In monkey, tracer injections into nIV or rostral nIII resulted in retrograde labeling of medium-sized GABAergic neurons in the contralateral INC (Horn et al., 2003). This is in line with the recordings of monosynaptic inhibitory postsynaptic potentials in nIV and nIII after INC stimulation (Schwindt et al., 1974). Recent findings in cat confirm these results, and re-emphasize that premotor inhibitory neurons in INC may represent inhibitory burst neurons of the vertical saccadic system (Sugiuchi et al., 2013).

### OCULOMOTOR SUBGROUPS INVOLVED IN HORIZONTAL GAZE

Aside from the CCN, the VEN, LAT, and DL subgroups in nIII receive a strong glycinergic input, as indicated by the relatively selective presence of GlyRs. In cat and monkey, glycinergic afferents were found to be associated specifically with motoneurons involved in *horizontal* eye movements, i.e., MR in nIII and lateral rectus muscle (LR) in the abducens nucleus (nVI). This is in contrast to the high concentration of GABAergic input to motoneurons for *vertical* eye movements in nIII and nIV (Spencer et al., 1989; Spencer and Baker, 1992). Tract-tracing experiments in monkey had shown that the MR is represented in two separated groups within nIII (Büttner-Ennever and Akert, 1981; Porter et al., 1983): the A-group occupying the ventral part of nIII and extending through its whole rostro-caudal extents, and the B-group forming a well separated DL group at caudal nIII levels (Büttner-Ennever and Akert, 1981). In addition at caudal levels, the MR population reaches as finger-like extensions into the fibers of the MLF, partly in conjunction with the A-group, partly forming completely separated islands. Based on the similar cytoarchitectural features and the selective glycinergic inputs, we consider the VEN group in human nIII as the homolog to the MR “A-group” in monkey, including the extensions of LAT into the surrounding MLF. Accordingly, the DL group is considered to be the homolog of the MR “B-group” (**Figure 8**): it has the same circular contour as in monkey, and a similar separation from the neighboring subgroups, with no motoneuronal dendrites extending beyond its boundaries. Interestingly, at the rostral nIII pole the dendrites of presumed MR motoneurons reach across the midline to their contralateral counterparts. Whether this is only the consequence of the disappearance of the NP at this level, or whether it has a functional background in the collection of common afferent inputs for controlling vergence, remains unclear.

The inhibitory control of horizontal gaze by glycinergic afferents that is seen for LR and MR motoneurons in cat and monkey is found to be preserved in the human as well (Spencer et al., 1989, 1992; Spencer and Baker, 1992). With anatomical, recording and pharmacological methods, the inhibitory nature of the glycinergic projection from the prepositus nucleus to the nVI has been demonstrated in the cat (Spencer et al., 1989). Up to date, the source of the glycinergic input to MR motoneurons in nIII is unknown. Although strychnine-sensitive GlyRs are known to mediate synaptic inhibition by activating chloride channels (Dutertre et al., 2012), glycine can also contribute to excitatory transmission by serving as an allosteric modulator for the glutamate N-methyl-D-aspartate receptor (Johnson and Ascher, 1987). Therefore, it is possible that the presence of GlyR seen in the MR subgroups in primates is associated with the glutamatergic inputs from the ipsilateral lateral vestibular nuclei via the ascending tract of Deiters (Nguyen and Spencer, 1999), which may contribute to viewing distance related gain changes of the vestibulo-ocular reflex (Snyder and King, 1992; Chen-Huang and McCrea, 1998).

However, in contrast to cat and monkey, in human all presumed MR subgroups receive an additional strong supply from GABAergic afferents, which even exceeds that of the motoneurons for vertical gaze. This finding is in line with observations from the human nVI, which also receives a strong GABAergic – in addition to a strong glycinergic – input. This observation is, surprisingly, *not* the same as in monkey, where only a moderate GABAergic input is observed (Spencer and Baker, 1992; Waldvogel et al., 2010). Thereby, the GABAergic inputs provided the least useful marker to delineate the motoneuronal subgroups in human nIII, but at the same time they revealed an interesting and unusual neuroanatomical difference between monkey and man, which is seldom observed.

Although the GABA-immunoreactivity in the cat nVI is relatively weak, both motoneurons and internuclear neurons get some GABAergic input (de la Cruz et al., 1989). In cat 20% of retrogradely labeled small internuclear neurons in and around the nIII expressed GABA-immunoreactivity (de la Cruz et al., 1992) and may be one source for the relatively weak GABAergic input to motoneurons and internuclear neurons in nVI (de la Cruz et al., 1992). In cat, tracer-labeled MR motoneurons receive a similar strong supply from glycinergic and GABAergic afferents (de la Cruz et al., 1992).

Up to date, it is generally accepted that horizontal conjugate eye movements are mediated through the nVI, which contains motoneurons and internuclear neurons. The motoneurons innervate the ipsilateral LR, the internuclear neurons activate the contralateral MR motoneurons in nIII via the MLF (for review: Horn and Leigh, 2011). A separate “extra-MLF” vergence pathway involving premotor neurons in the supraoculomotor area (SOA) with pure vergence signals (not conjugate eye movements) provides the command to move the eyes at equal magnitudes, but in opposite direction for alignment of gaze between targets at different depths (Mays, 1984). At the same location in the SOA, divergence neurons have been identified, which showed decreased firing rates with increasing vergence angles (Mays, 1984; Judge and Cumming, 1986). Direct inputs from the SOA to MR motoneurons have been demonstrated (Zhang et al., 1991), and they were shown to be related either to pure vergence or accommodation, or to both (Zhang et al., 1992). Theoretically, divergent eye movements require the activation of LR motoneurons and inhibition of MR motoneurons, which could be mediated through inhibition from GABAergic neurons in the SOA.

Another direct premotor input to motoneurons of the horizontal system was indicated from the central mesencephalic reticular formation (CMRF) after retrograde transsynaptic labeling studies in monkey applying rabies virus injections into LR (Ugolini et al., 2006; Büttner-Ennever, 2008). The CMRF is closely interconnected with the superior colliculus and the paramedian pontine reticular formation including the saccadic omnipause neurons (Cohen and Büttner-Ennever, 1984; Chen and May, 2000; Wang et al., 2013) and has been found to be correlated with horizontal and vertical saccades (Waitzman et al., 2000a,b, 2002). Preliminary data applying small biotin dextran injections into the rostromedial part of the CMRF in monkey revealed monosynaptic inputs to all MR motoneuron subgroups on both sides and pg neurons in the EWpg, indicating a role in vergence and the near triad, at least of this CMRF region (May et al., 2011; Horn et al., 2012). The accompanying ultrastructural analysis revealed that many of the tracer labeled terminals contacting MR motoneurons have features in accordance with inhibitory synapses, some of them expressing GABA-immunolabeling (May et al., 2011). To what extent the GABA-negative afferent terminals may represent glycinergic afferents remains to be studied. Based on the monkey data, the strong GABAergic input seen here in the human nIII may derive at least in part from the adjacent CMRF and/or SOA.

### GENERAL ORGANIZATION IN OCULOMOTOR NUCLEUS: PRIMATE

The first anatomical description of the nIII is given by Stilling (1846). The partition into a dorsal and ventral portion, and the presence of numerous decussating axons was first described by von Gudden (1881; for review: Warwick, 1953a). A very precise description of the cytoarchitecture of the nIII was provided by Perlia on fetal human brain, which included the lateral and medial portion of the classical EW and the NP, which he originally had termed “Centralkern” (Perlia, 1889). Based on observations made after the removal of extraocular muscles in various species, different variations of an nIII map had been proposed (reviewed by Warwick, 1953a). The elaborate work of Warwick, who plotted the neurons undergoing chromatolysis after the resection of individual extraocular muscles in monkey, provided a map of the primate nIII, which was widely accepted and used as basis for the human nIII in many textbook illustrations (Warwick, 1953a). The organization of the motoneuronal groups shows a sequence from rostral to caudal of IR, MR, IO, SR, and LP motoneurons. The newly developed tract-tracing method basically confirmed the proposed arrangement of motoneuronal groups of individual muscles in the nIII of monkey, but it revealed for the first time the presence of two motoneuron groups for the MR, the ventral A-group and the DL circular B-group (Büttner-Ennever and Akert, 1981; Porter et al., 1983; Büttner-Ennever, 2006). This two-fold representation of the MR within the nIII is most evident in primates and its function remains unclear (Augustine et al., 1981; Sun and May, 1993; Büttner-Ennever, 2006). So far no differences in histochemistry or afferent inputs have been found between the A- and B-group (Spencer et al., 1992; Wasicky et al., 2004; Erichsen et al., 2014).

It has been known for a long time that extraocular muscles exhibit a complex architecture consisting of a global and orbital layer. At least six different types of muscle fibers can be identified, which can be divided into two main categories of SIF and multiply-innervated non-twitch muscle fibers (MIF; for review: Spencer and Porter, 2006). Tract-tracing experiments in monkey revealed that the motoneurons of MIFs are located in the periphery of the motonuclei. For muscles innervated from the nIII the MIF motoneurons of IR and MR are located in the C-group DM to nIII, and those of IO and SR in the S-group between the both nIII (Büttner-Ennever et al., 2001). Based on their different histochemical properties, SIF motoneurons were identified within nIII and putative MIF motoneurons have been identified around the medial aspects of nIII, also in human (Eberhorn et al., 2005, 2006). However, in the human nIII, the MIF motoneurons could not be allocated to specific extraocular muscles, yet (Horn et al., 2008). Therefore, the proposed map of the human nIII applies only to the SIF motoneurons within nIII, and has yet to be extended in future studies by the location of MIF motoneurons.

The exact knowledge of the location of the subgroups innervating individual eye muscles in human provides an important basis to localize lesions more accurately in MRI scans and relate it to clinical findings. Furthermore, the present work on transmitter inputs to individual eye muscle subgroups will form the basis for postmortem studies of afferent inputs to nIII in cases with known eye-movement deficits

## AUTHOR CONTRIBUTIONS

Acquisition of data and analysis was performed by Emmanuel Che Ngwa, Christina Zeeh, and Ahmed Messoudi. Conception of the work was done by Anja K. E. Horn and Jean A. Büttner-Ennever. All authors contributed to the interpretation of data, preparation of the figures, and writing of the manuscript and approved the final version.

## Conflict of Interest Statement

The authors declare that the research was conducted in the absence of any commercial or financial relationships that could be construed as a potential conflict of interest.

## Abbreviations

nIII: oculomotor nucleus
nIV: trochlear nucleus
nVI: abducens nucleus
CCN: central caudal nucleus
CEN: central group
ChAT: choline acetyltransferase
CMRF: central mesencephalic reticular formation
CR: calretinin
DL: dorsolateral group
DM: dorsomedial group
DR: dorsal raphe nucleus
EAP: extravidin-peroxidase
EW: Edinger–Westphal nucleus
EWcp: centrally projecting Edinger–Westphal nucleus
EWpg: Edinger–Westphal nucleus containing preganglionic neurons
GABA: gamma-aminobutyric acid
GAD: glutamate decarboxylase
GlyR: glycine receptor
IC: inferior colliculus
INC: interstitial nucleus of Cajal
IO: inferior oblique muscle
IPN: interpeduncular nucleus
IR: inferior rectus muscle
LP: levator palpebrae muscle
LR: lateral rectus muscle
MGB: medial geniculate body
MIF: multiply-innervated non-twitch muscle fibers
ML: medial lemniscus
MLF: medial longitudinal fasciclus
MR: medial rectus muscle
NIII: oculomotor nerve
NP: nucleus of Perlia
NP-NF: non-phosphorylated neurofilaments
PAG: periaqueductal gray
PB: phosphate buffer
PN: pontine nuclei
RIMLF: rostral interstitial nucleus of the medial longitudinal fasciculus
RN: red nucleus
SC: superior colliculus
SCP: superior cerebellar peduncle
SE of mean: standard error of the mean
SIF: singly-innervated twitch muscle fibers
SNc: substantia nigra pars compacta
SNr: substantia nigra pars reticulata
SO: superior oblique muscle
SOA: supraoculomotor area
SR: superior rectus muscle
UCN: urocortin
VEN: ventral group
